# Integrative cfDNA profiling from low-pass whole-genome sequencing enables tissue-of-origin prediction in cancer

**DOI:** 10.1186/s43556-026-00497-2

**Published:** 2026-08-05

**Authors:** Yunjian Zhang, Liang Liu, Hua Bao, Ke Xu, Hao Zhang, Song Wang, Shuang Chang, Dongqin Zhu, Zongyao Huang, Zheng Wang, Liu Yang, Bingzhong Zhang, Ji Tao, Wenhua Liang, Jierong Chen, Shanshan Yang, Xue Wu, Yang Shao, Wenquan Wang, Dongyuan Zhu

**Affiliations:** 1 Department of Breast Surgery, First Affiliated Hospital of Sun Yat-sen University, Guangzhou, China; 2 Department of Pancreatic Surgery, Zhongshan Hospital, Fudan University, Shanghai, China; 3 Geneseeq Research Institute, Nanjing Geneseeq Technology Inc., Nanjing, China; 4 Thoracic Surgery Laboratory, Xuzhou Medical University, Xuzhou, China; 5 Department of Thoracic Surgery, Affiliated Hospital of Xuzhou Medical University, Xuzhou, China; 6 Department of Pathology, Sichuan Clinical Research Center for Cancer, Sichuan Cancer Hospital & Institute, Sichuan Cancer Center, University of Electronic Science and Technology of China, Chengdu, China; 7 Department of Liver Surgery and Transplantation, Liver Cancer Institute, Zhongshan Hospital, Fudan University, Shanghai, China; 8 Colorectal Center, Jiangsu Cancer Hospital, Nanjing, China; 9 Department of Gynecologic Oncology, Sun Yat-sen Memorial Hospital, Sun Yat-sen University, Guangzhou, China; 10 Department of Gastrointestinal Medical Oncology, Harbin Medical University Cancer Hospital, Harbin, China; 11 Department of Thoracic Surgery and Oncology, The First Affiliated Hospital of Guangzhou Medical University, Guangzhou, China; 12 Department of Clinical Laboratory, Guangdong Provincial People’s Hospital, Guangzhou, China; 13 School of Public Health, Nanjing Medical University, Nanjing, China; 14 Department of Rare Tumors, Shandong Cancer Hospital and Institute, Shandong First Medical University and Shandong Academy of Medical Sciences, Jinan, China

**Keywords:** Multi-cancer classification, cfDNA profiling, Whole genome sequencing, Tissue-of-origin

## Abstract

Cancer type classification is challenging due to tumor heterogeneity and undefined tissue of origin (TOO), particularly in cancers of unknown primary (CUP) and multiple primary cancers (MPC). Accurate TOO identification is critical for guiding treatment and prognosis. We developed a stacked ensemble machine learning classifier that integrates 11 multidimensional cfDNA features spanning genomic, fragmentomic, methylation/repeat, and microbial signals. Base models were constructed using five algorithms, including Deep Learning, Distributed Random Forest, Gradient Boosting Machine, Generalized Linear Model, and XGBoost, within a five-fold cross-validation framework, and their predictions were aggregated into a final ensemble optimized for top-1 accuracy. The classifier achieved robust performance across 17 cancer types, with top-1 and top-2 accuracies of 78% and 89% in the training cohort (*n* = 1,814), and 80% and 90% in an independent validation cohort (*n* = 1,221). Notably, predictive performance was retained in samples with low tumor fraction (71% top-1, 85% top-2). Sensitivity varied across tumor types, with the highest performance observed in head and neck and colorectal cancers. Among CUP cases, 11 of 15 (73.3%) predictions matched clinically inferred primary sites based on multimodal diagnostics. Feature importance analysis identified nucleosome positioning, fragment size distribution, and repeat elements as key contributors to model performance. Collectively, this cfDNA-based classifier provides a robust and non-invasive approach for accurate cancer type identification and has the potential to support clinical decision-making.

**Supplementary Information:**

The online version contains supplementary material available at 10.1186/s43556-026-00497-2.

## Introduction

Cancer is the second leading cause of death worldwide, and its burden is expected to increase due to population growth and aging [1]. Accurate classification of cancer by its tissue of origin (TOO) or primary site is critical for guiding clinical therapeutic strategies, particularly because many precision treatments are tumor-type specific [2–4]. However, conventional diagnostic approaches, such as imaging and biopsies, are often hampered by tumor heterogeneity and the inaccessibility of certain tumor sites. This predicament is especially pronounced in cancers of unknown primary (CUP), which account for 3–5% of all malignancies [5], where the primary site remains undetermined despite comprehensive clinical evaluation [6]. Biopsies are invasive, may cause patient discomfort, and carry a risk of complications, highlighting the need for accurate and non-invasive methods to determine cancer origin.

Recent advances in liquid biopsy using plasma cell-free DNA (cfDNA) have shown potential in cancer detection and monitoring treatment response [7, 8]. cfDNA refers to short DNA fragments released into the circulation by apoptotic or necrotic cells. Its epigenetic signatures are closely associated with TOO, providing insights for tissue deconvolution [9, 10]. A subset of cfDNA, known as circulating-tumor DNA (ctDNA), derived from tumor cells, carries tumor-specific molecular signals that can be leveraged for cancer classification and TOO prediction [11]. Several cfDNA-based classifiers have been developed based on genomic profiling or DNA methylation patterns [5, 12–14], while recent studies on cfDNA fragmentomics demonstrate that fragmentation patterns differ between healthy individuals and cancer patients, reflecting underlying epigenetic and transcriptional changes [15, 16]. However, most existing TOO models rely on a limited set of cfDNA features, leaving many informative fragmentation-related signals largely unexplored.

Whole-genome sequencing (WGS)-based cfDNA profiling captures diverse molecular landscapes. Beyond genetic mutations and structural variants, WGS detects fragmentation features shaped by nucleosome positioning, CpG methylation, transcription factor binding, and other chromatin-associated processes. These signatures provide multidimensional biological information, which holds promise for improving cancer classification and TOO prediction. To date, no model has comprehensively integrated all these features. Existing classifiers also show reduced performance in cfDNA samples with low tumor fraction (TF) [17], and their applicability to cases of multiple primary cancers (MPC), defined as the occurrence of more than one synchronous or metachronous cancer in the same individual [18], remains largely unexplored. In certain cancers, such as cervical cancer driven by viral infection, viral sequences detectable in cfDNA represent additional oncogenic signals that can be incorporated into classification [19].

Here, we present a machine learning model that integrates 11 categories of cfDNA features derived from low-pass WGS to predict the primary cancer sites across 17 cancer types. The model was developed using a large dataset and incorporates diverse fragmentomic-based cfDNA features spanning genomic, fragmentation patterns, methylation/repeat profiles, and microbial signals. Its performance was validated in an independent cohort and further evaluated in exploratory cohorts of CUP and MPC. We also assess its feasibility in patient samples with low tumor fractions, underscoring its potential clinical utility in diagnostically challenging cases.

## Results

### Dataset preparation and feature extraction

We assembled a training cohort of 1,814 participants and an independent validation cohort of 1,221 participants, covering 17 cancer types, including breast, cervical, colorectal, esophageal, gastric, head and neck, kidney, liver/biliary, lung, ovarian, pancreatic, prostate, thyroid, urothelial, uterine, melanoma, and sarcoma (Table 1). Baseline demographics and cancer type distribution were comparable between cohorts.

**Table 1:** **C**linical characteristics of the training and validation cohort

	**Training (*N* = 1,814)**	**Validation (*N* = 1,221)**	***P*-value**
**Age**			0.812
Median (IQR)	60 (52–68)	60 (52–68)	
≤ 60 years	934 (51.5%)	625 (51.2%)	
> 60 years	819 (45.2%)	558 (45.7%)	
Unknown	61 (3.4%)	38 (3.1%)	
**Sex**			0.627
Male	1006 (55.5%)	539 (44.1%)	
Female	784 (43.2%)	665 (54.5%)	
Unknown	24 (1.3%)	17 (1.4%)	
**Stage**			0.308
0	10 (0.6%)	0 (0%)	
I	445 (24.5%)	157 (12.9%)	
II	308 (17.0%)	137 (11.2%)	
III	334 (18.4%)	314 (25.7%)	
IV	417 (23.0%)	413 (33.8%)	
Unknown	300 (16.5%)	200 (16.4%)	
**Cancer type**			0.703
Breast	125 (6.9%)	80 (6.6%)	
Cervical	97 (5.3%)	63 (5.2%)	
Colorectal	137 (7.6%)	94 (7.7%)	
Esophageal	76 (4.2%)	52 (4.3%)	
Gastric	155 (8.5%)	105 (8.6%)	
Head and neck	101 (5.6%)	71 (5.8%)	
Kidney	76 (4.2%)	56 (4.6%)	
Liver/Biliary	194 (10.7%)	131 (10.7%)	
Lung	160 (8.8%)	108 (8.8%)	
Ovarian	127 (7.0%)	82 (6.7%)	
Pancreatic	107 (5.9%)	72 (5.9%)	
Prostate	111 (6.1%)	74 (6.1%)	
Thyroid	59 (3.3%)	38 (3.1%)	
Urothelial	80 (4.4%)	54 (4.4%)	
Uterine	73 (4.0%)	50 (4.1%)	
Melanoma	50 (2.8%)	34 (2.8%)	
Sarcoma	86 (4.7%)	57 (4.7%)	

*IQR* interquartile range

**Fig. 1:**
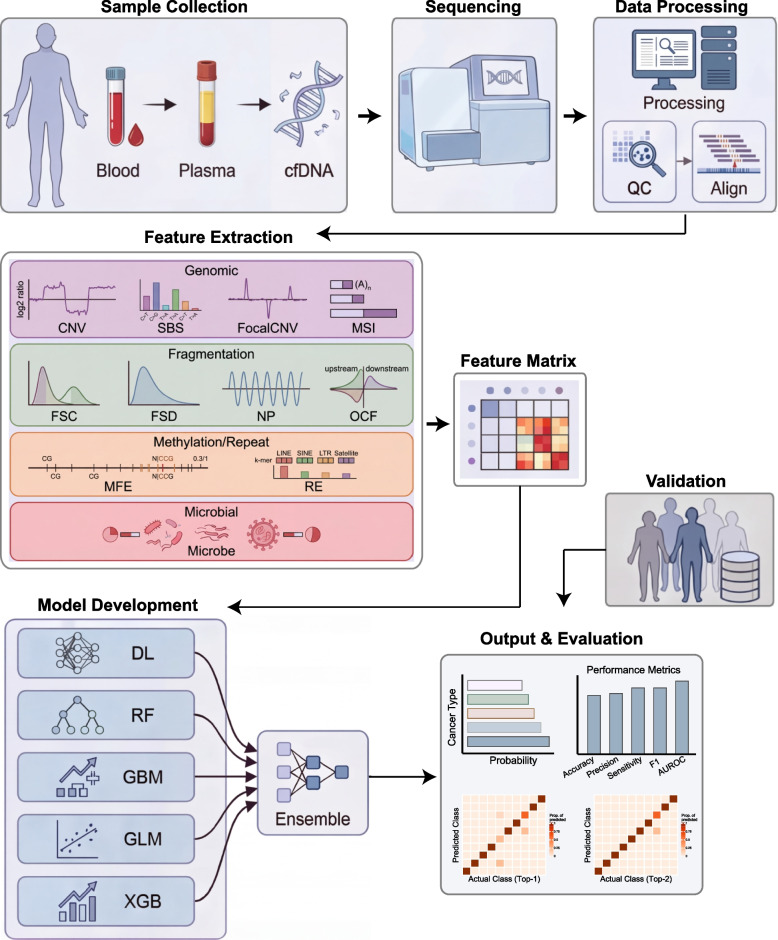
Development of a cfDNA-based machine learning model for multi-cancer classification. Schematic overview of the study design. Peripheral blood samples were collected from cancer patients, followed by plasma isolation and cfDNA extraction. Samples were analyzed using low-pass whole-genome sequencing and processed through a standardized bioinformatics pipeline. A diverse set of cfDNA features was extracted, including genomic features (CNV, SBS, FocalCNV, MSI), fragmentation features (FSC, FSD, NP, and OCF), methylation/repeat element features, and microbial signatures. These features were compiled into a feature matrix for model development. Base models were trained using five machine learning algorithms: deep learning (DL), Random Forest (RF), Gradient Boosting Machine (GBM), Generalized Linear Model (GLM), and XGBoost, and were subsequently combined into a stacked ensemble model. Model performance was evaluated in independent validation cohorts based on the predicted probabilities for each cancer type. Abbreviations: CNV, copy number variation; SBS, SBS-based mutational signatures; FocalCNV, focal somatic copy number alterations; MSI, microsatellite instability; FSC, fragment size coverage; FSD, fragment size distribution; NP, nucleosome profiling; OCF, orientation-aware cfDNA fragmentation. AUROC, area under the receiver operating characteristic curve

cfDNA was extracted from blood samples and sequenced using low-pass WGS at 5 × coverage. Eleven cfDNA feature groups capturing complementary biological and structural characteristics were analyzed and classified into four types (Fig. 1). Genomic features, including copy number variation (CNV), focal somatic copy number alterations (FocalCNV), single base substitution signatures (SBS), and microsatellite instability (MSI), reflect DNA copy number changes and mutational patterns. Fragmentation features, comprising fragment size coverage (FSC), fragment size distribution (FSD), nucleosome profiling (NP), and orientation-aware cfDNA fragmentation (OCF), capture cfDNA length distributions, nucleosome positioning, chromatin accessibility, and fragment orientation patterns. Methylation and repeat features, represented by methylation-associated fragment ends (MFE) and repeat elements (RE), characterize DNA methylation-associated fragment ends and repetitive sequence composition. Microbial features, representing microbial DNA sequences, quantify pathogen-derived cfDNA fragments.

To assess the discriminative capacity of these features, we first performed supervised dimensionality reduction using linear discriminant analysis (LDA). Projections based on the full feature set revealed clear separation across cancer types in both the training and validation cohorts (Fig. S1a, b).

We then conducted a preliminary evaluation comparing individual feature categories (excluding MSI) against the integrated multi-feature model (“All features”). MSI was excluded from this comparison because it is represented as a single continuous score per sample, whereas other feature categories comprise high-dimensional feature vectors, making direct comparison less informative. No single feature category achieved performance comparable to the combined model; integrating multiple features consistently improved predictive performance across both cohorts (Fig. S2a, b). These results suggest that different cfDNA feature groups capture complementary biological signals across multiple molecular and structural dimensions. Although not included in the comparative analysis, MSI is a well-established genomic marker with known relevance in a subset of cancer types. Consequently, all 11 cfDNA feature groups, including MSI, were retained and incorporated into the final machine learning model for cancer type classification.

### Model performance on the training dataset

**Fig. 2:**
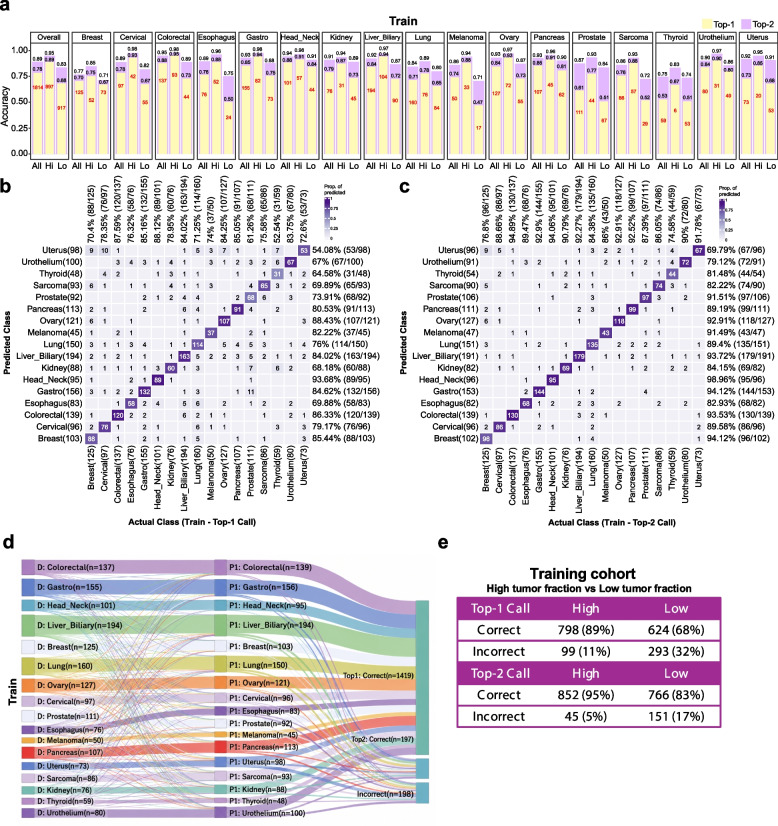
Model performance in the training cohort. **a** Bar plots showing the accuracy of the multi-cancer classifier across 17 cancer types in the training cohort. Samples are stratified by tumor fraction: high (Hi; ctDNA ≥ 3%) and low (Lo; ctDNA < 3%). Total sample counts per group are indicated in red, and the percentage of correctly predicted samples is shown in black. Purple bars represent “top-1” accuracy, and yellow bars represent “top-2” accuracy (samples correctly classified within the two most likely types). **b**, **c** Confusion matrices illustrating top-1 (b) and top-2 (c) prediction results across 17 cancer types. Rows represent predicted classes and columns represent actual clinical diagnoses. Cell values indicate sample counts, with color intensity reflecting the proportion of predictions. Diagonal values correspond to correctly classified samples. Per-class sensitivity is indicated on the top horizontal axis, and per-class precision is indicated on the right vertical axis. **d** Alluvial plot illustrates the mapping between the actual clinical diagnoses (left, D) and the top-1 predicted cancer types (right, P1). Each horizontal flow (ribbon) represents a group of samples, with the ribbon width proportional to the number of samples. Ribbons flowing directly across to the same category indicate correct predictions, while diverging ribbons highlight specific misclassifications.** e** Table summarizing the model’s accuracy and error rates across high and low tumor fraction samples in the training cohort

We first evaluated the diagnostic performance of the classifier in the training cohort (*n* = 1,814). Overall, the model demonstrated robust performance, achieving an accuracy of 78% and a macro-F1 score of 0.767 (Fig. 2a; Table 2). It also exhibited strong discriminative power across all classes, with a weighted-AUROC of 0.973.

**Table 2:** Model performance metrics for top-1 predictions in the training and validation cohorts

Performance metrics	Train	Valid
Accuracy	0.782	0.799
Macro_precision	0.769	0.789
Macro_sensitivity	0.770	0.783
Macro_F1	0.767	0.784
Macro_AUROC	0.971	0.973
Weighted_precision	0.788	0.801
Weighted_sensitivity	0.782	0.799
Weighted_F1	0.783	0.799
Weighted_AUROC	0.973	0.974

A more detailed analysis across the 17 cancer types revealed variable classification performance (Fig. 2b; Table S1). Several common malignancies showed high sensitivity and precision; for instance, head and neck cancer achieved a sensitivity of 0.881 (89/101), while colorectal cancer reached 0.876 (120/137). In contrast, more challenging categories such as thyroid and prostate cancers exhibited lower top-1 sensitivities of 0.525 and 0.613, respectively. Notably, AUROC values for these cancer types remained high (thyroid: 0.940; prostate: 0.966), suggesting that the captured relevant biological signals were maintained despite reduced categorical accuracy.

We next evaluated the incremental benefit of incorporating the model’s second-highest prediction. Across the training cohort, the top-2 accuracy reached 0.89, representing an 11% absolute increase over the top-1 accuracy (Fig. 2a-c). This gain was particularly pronounced in cancer types with low top-1 performance; for example, in thyroid cancer, accuracy increased from 0.53 to 0.75, and in prostate cancer, from 0.61 to 0.87. In several cancer types, top-2 accuracy approached near-saturation levels, including colorectal (0.95), head and neck (0.94), gastro (0.93), and liver_biliary (0.92). This high top-2 performance provides a clinically meaningful “diagnostic buffer”, narrowing the differential diagnosis and reducing the risk of misdirecting downstream site-specific workups. Overall prediction patterns were visualized using an alluvial plot (Fig. 2d).

To assess the impact of ctDNA abundance, samples were stratified into low (< 3%) and high (≥ 3%) tumor fraction groups. The classifier achieved top-1 and top-2 accuracies of 89% and 95%, respectively, in the high-TF group, compared with 68% and 83% in the low-TF group (Fig. 2e; Fig. S3). These results highlight ctDNA abundance as a key determinant of classification performance, consistent with increased tumor-derived signal in high TF samples. Despite reduced sensitivity in low-TF samples, the model retained reasonable predictive ability for several cancer types, including head and neck (84%), pancreatic (81%), and urothelial cancers (80%) (Fig. S3c). In contrast, sensitivity was relatively lower for esophageal, melanoma, prostate, and sarcoma cancers, suggesting that these tumor types may release less ctDNA or exhibit less distinctive molecular signals at low tumor burden.

### Model performance on the independent validation dataset

**Fig. 3:**
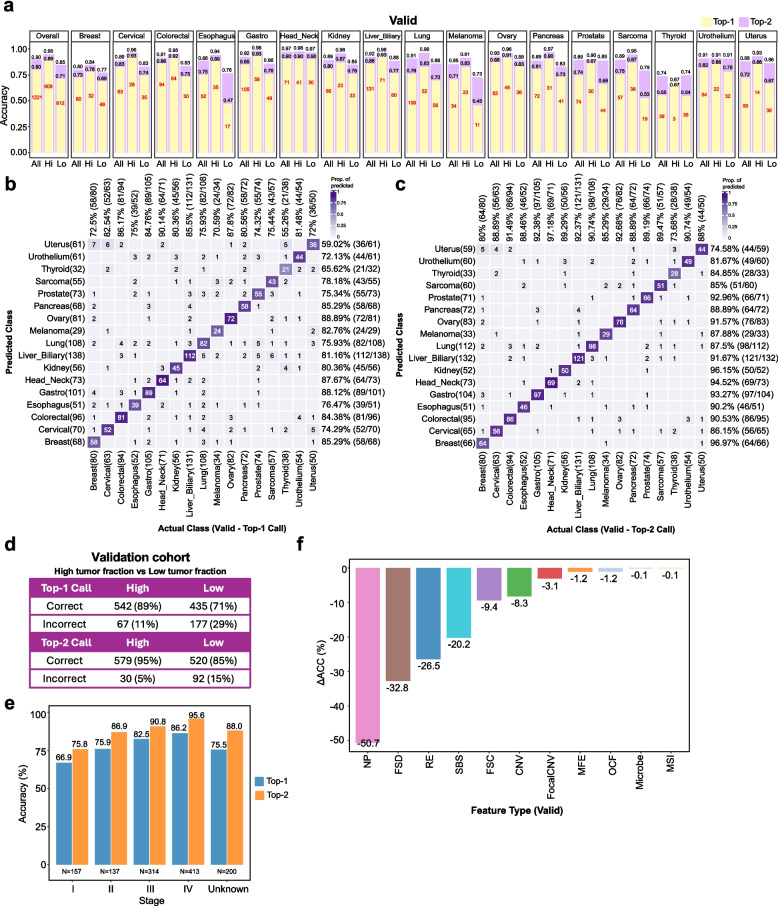
Model performance in the validation cohort. **a** Bar plots showing the accuracy of the multi-cancer classifier across 17 cancer types in the independent validation cohort. Samples are stratified by tumor fraction: high (Hi; ctDNA ≥ 3%) and low (Lo; ctDNA < 3%). Total sample counts per group are indicated in red, and the percentage of correctly predicted samples is shown in black. Purple bars represent “top-1” accuracy, and yellow bars represent “top-2” accuracy (samples correctly classified within the two most likely types). **b**, **c** Confusion matrices illustrating top-1 (b) and top-2 (c) prediction results across 17 cancer types. Rows represent predicted classes and columns represent actual clinical diagnoses. Cell values indicate sample counts, with color intensity reflecting the proportion of predictions. Diagonal values correspond to correctly classified samples. Per-class sensitivity is indicated on the top horizontal axis, and per-class precision is indicated on the right vertical axis. **d** Table summarizing the model’s accuracy and error rates across high and low tumor fraction samples in the validation cohort. **e** Accuracy of top-1 (blue) and top-2 (orange) predictions across different cancer stages. **f** Bar plot depicting the contribution of the 11 cfDNA feature sets to the classifier. Importance is measured by the change in accuracy (ΔACC%) after removing each specific feature group

We evaluated our classifier on the independent validation cohort (*n* = 1,221). The model achieved an overall accuracy of 80% for top-1 predictions and 90% for top-2 predictions, with consistently high sensitivity across all cancer types (Fig. 3a-c; Table S2). Consistent with the training cohort, thyroid cancer showed the lowest sensitivity (0.553), whereas head and neck cancer achieved the highest sensitivity for both top-1 and top-2 predictions (0.901 and 0.972). Performance remained strong across ctDNA levels, correctly classifying 89% (542/609) of high-TF and 71% (435/612) of low-TF samples (Fig. 3d; Fig. S4).

We then examined classifier performance across clinical stages, observing that accuracy increased with disease progression, from 66.8% (stage I) to 86.2% (stage IV) for top-1 predictions, and from 75.8% to 95.6% for top-2 predictions, likely reflecting increasing ctDNA abundance in advanced disease (Fig. 3e). To assess potential influence of ctDNA abundance, we performed stratified analyses, which revealed consistently higher performance in samples with high TF across all stages (Fig. S5). Although performance was lower in low-TF samples, the classifier remained discriminative across both stage and TF subgroups. In stage I samples with high TF, the model achieved a macro-averaged AUROC of 94.1% and a weighted-averaged AUROC of 96.5%. Across low-TF stage subgroups, weighted-averaged AUROC remained above 91%. These findings suggest that higher ctDNA abundance improves performance, but tissue-associated signals remain informative across different stages and tumor burdens.

Feature contribution analysis identified NP as the most critical feature, with its removal reducing overall accuracy by 50.7%, followed by FSD (32.8%) and RE (26.5%) (Fig. 3f). In contrast, microbial features and MSI contributed the least, likely reflecting their limited prevalence across the cohort, which may lead to an underestimation of their overall contribution (Fig. S6). Nevertheless, these features may still provide critical and highly specific information in particular cancer types or biological contexts.

### Application of the classifier in cancers of unknown primary

**Fig. 4:**
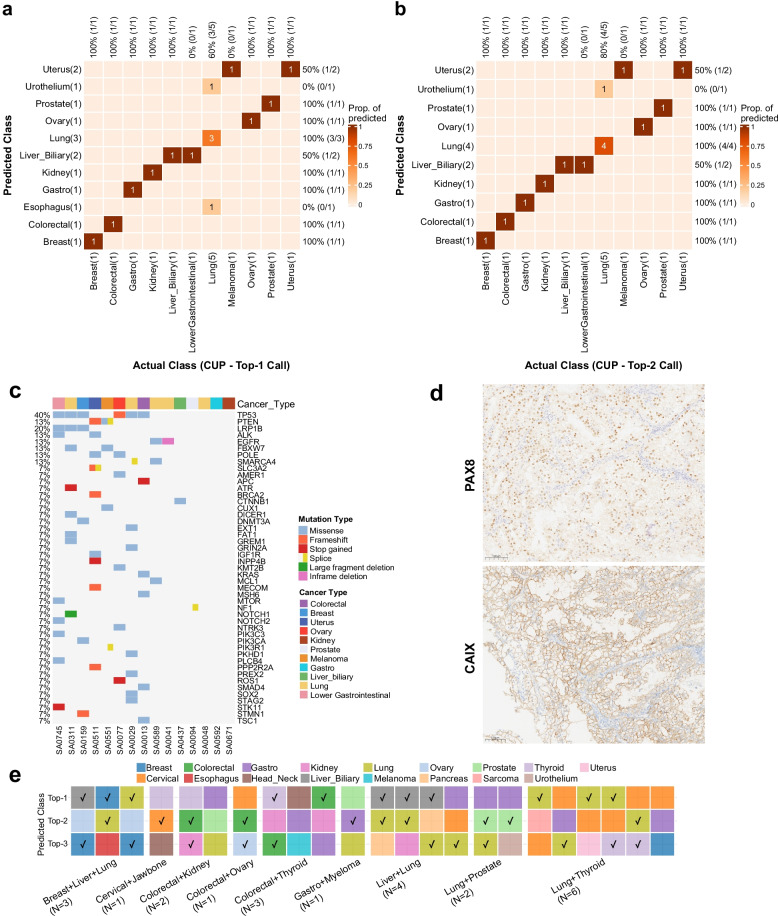
Exploratory evaluation of the model performance in CUP and MPC cohorts. **a**, **b** Confusion matrices showing top-1 (**a**) and top-2 (**b**) predictions for cancer of unknown primary (CUP) samples. Rows represent predicted classes and columns represent actual clinical diagnoses. Cell values indicate sample counts, with color intensity reflecting the proportion of predictions. Diagonal values correspond to correctly classified samples. Per-class sensitivity is indicated on the top horizontal axis, and per-class precision is indicated on the right vertical axis. **c** Oncoprint showing the mutational landscape of patients in the CUP cohort. **d** Representative immunohistochemistry staining of PAX8 (top) and CAIX (bottom) in lymph node samples from a CUP patient. **e** Prediction summary for the multiple primary cancer (MPC) cohort. Each column represents an individual patient, and rows correspond to the top-1, top-2, and top-3 predicted cancer types (color-coded). Checkmarks indicate predictions concordant with clinical diagnoses

CUP represents a subset of metastatic malignancies in which the primary cancer site cannot be determined through standard diagnostic work-up, often due to atypical metastatic patterns, occult or regressed primary tumors, and the absence of detectable lesions. As a result, CUP patients typically have a poor prognosis [20]. Several CUP classifiers had been developed, including RNA-, DNA- [5, 12, 21, 22] and methylation-based [13, 23] approaches using cfDNA profiling. However, none incorporate fragmentation patterns. To evaluate the clinical utility of our classifier, we analyzed 15 CUP patients with clinically inferred primary sites based on comprehensive histopathological assessment, including tumor morphology and immunohistochemistry (IHC), together with available clinical information (Supplementary Material 2). Our model correctly predicted the primary site in 11 of 15 cases, spanning 11 cancer types, demonstrating strong agreement with clinical diagnoses (Fig. 4a, b). To further support classification, all CUP samples underwent targeted sequencing of 437 cancer-related genes. Detected mutation profiles were consistent with the predicted cancer types, providing orthogonal validation of model predictions (Fig. 4c).

A representative case highlights the clinical relevance of our approach. Patient SA0671 presented with persistent back pain, and PET-CT revealed widespread lymphadenopathy and hypermetabolic lesions, initially suggestive of lymphoma. However, biopsy identified poorly differentiated metastatic carcinoma. IHC staining showed positivity for PAX8 and CAIX, markers strongly associated with renal cell carcinoma, supporting a kidney origin (Fig. 4d) [24, 25]. The patient was subsequently diagnosed with clear cell renal cell carcinoma. Consistently, our model correctly identified the kidney as the top predicted tissue of origin.

Together, these results demonstrate that our model can provide clinically meaningful, complementary evidence for CUP diagnosis, potentially improving diagnostic confidence and guiding treatment decisions.

### Exploratory evaluation of the classifier in patients with multiple primary cancers

MPC is defined as the occurrence of two or more primary tumors in a single individual, either synchronously or metachronously, arising from different anatomic sites or histological origins [26]. Diagnosis typically relies on histopathology and imaging modalities such as PET-CT [27]. However, repeated biopsies are invasive, and some lesions can be inaccessible or unsuitable for sampling, highlighting the need for non-invasive molecular approaches.

Given that cfDNA in MPC patients may originate from multiple tumor sources, we sought to investigate how the classifier behaves in the presence of potentially mixed signals, which represents a clinically relevant but challenging scenario. To this end, we retrospectively analyzed 23 MPC patients with clinically confirmed diagnoses based on comprehensive histological and imaging evaluation following the National Cancer Institute’s Surveillance, Epidemiology and End Results (NCI SEER) criteria. In patients with two primary tumors, both cancer types were captured within the top three predicted probabilities in 9 of 20 cases, while in patients with three primary tumors, two of the three cancer types were identified within the top three predictions (Fig. 4e).

**Fig. 5:**
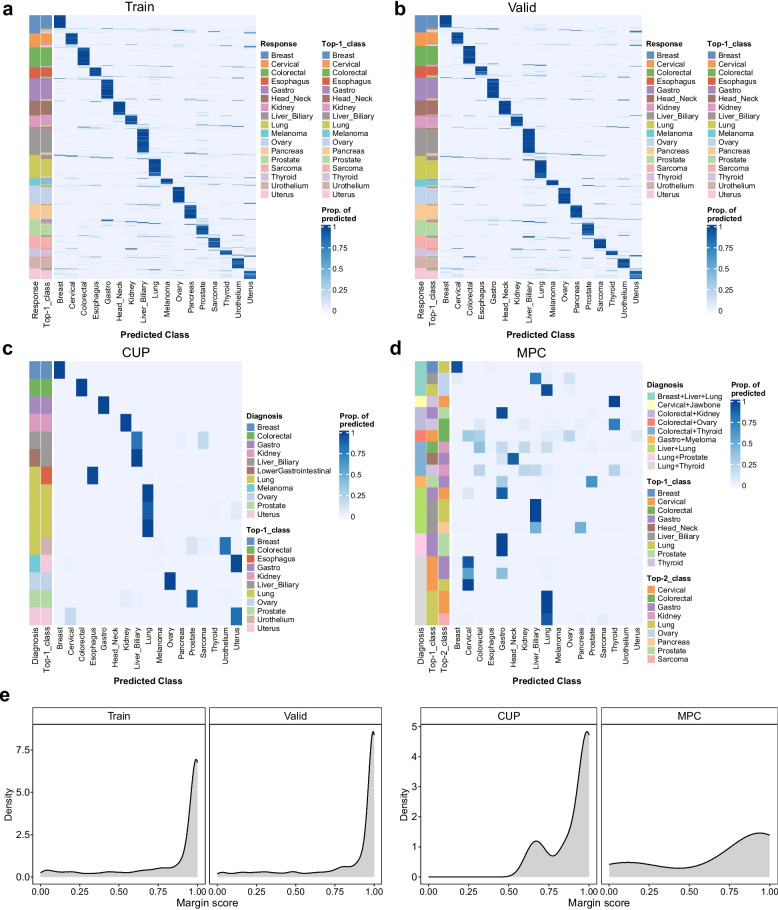
Model classification patterns and distribution of margin scores across cohorts. **a-d** Heatmaps displaying the predictive patterns for the train (**a**), validation (**b**), cancers of unknown primary (**c**), and multiple primary cancers (**d**) cohorts. **e** Density plots of the margin score for each cohort. The margin score is defined as the numerical difference between the top-1 and top-2 prediction probabilities

To support clinical interpretation of model predictions, we evaluated the margin score, defined as the probability difference between the top two predictions (Fig. 5). In the training and validation cohorts, most samples exhibited high margin scores with clear separation between predicted tissue types, while the CUP cohort showed predominantly moderate-to-high margin scores, indicating a generally dominant tissue-of-origin signal (Fig. 5a, b, e). In contrast, MPC samples more frequently showed reduced margin scores and flatter predicted distributions, reflecting the presence of mixed cfDNA contributions. High margin scores in MPC occurred when one tumor dominated the cfDNA signal, whereas lower margin scores were observed when multiple tumors contributed more evenly. These observations highlight the complexity of interpreting cfDNA signals in MPC cases. Compared with the relatively clear separation observed in training, validation, and CUP cohorts, the less distinct prediction patterns in MPC underscore the limitations of the current classifier in this context. These findings should be interpreted cautiously and in conjunction with clinical, radiological, and pathological information, and may inform the development of future models designed to accommodate multiple tumor origins.

## Discussion

Accurate TOO classification remains a critical challenge in cancer management, particularly in the era of precision medicine, where therapeutic strategies are increasingly tailored to cancer type and molecular characteristics. Plasma cfDNA provides a non-invasive approach to capture tumor-derived information, as it carries rich, nonrandom fragmentation patterns that reflect TOO signals. Leveraging low-pass WGS and integrative bioinformatics, our study captures multiple complementary dimensions of cfDNA biology, facilitating non-invasive prediction of tumor origin to guide clinical decision-making. Importantly, this model is intended to complement, rather than replace, existing diagnostic strategies and is applied in contexts where cancer is already suspected or confirmed, directly informing diagnostic workup, particularly for patients with CUP.

Unlike existing WGS-based classifiers that focus on individual feature types, our model integrates genomic, fragmentomic, methylation/repeat, and microbial signals, providing a multidimensional representation of cfDNA. Fragmentomic features, such as NP and FSD, form the structural backbone of the classifier, while genomic features (SBS, CNV, and FocalCNV) refine classification in mutationally rich tumors. Microbial features and MSI, although contributing less overall, serve as high-specificity tiebreakers in virus-associated or MSI-H malignancies.

Compared with prior approaches, our model demonstrates several advantages. Methylation-based assays, such as Galleri (GRAIL) [28], report high accuracy (88.7%) for cancer signal origin prediction; however, this performance depends on prior identification of positive samples by a preceding model with limited sensitivity (51.5%), thereby excluding a substantial proportion of low-signal or more challenging cases. In addition, bisulfite conversion compromises DNA integrity and library complexity, potentially reducing performance in samples with low ctDNA abundance. Fragmentomics-based methods, such as DELFI [29], achieve top-1 accuracies of 44%−76% across seven cancer types by capturing cfDNA fragment length patterns, but are limited by reliance on a single feature type and restricted cancer coverage. Multi-analyte approaches like CancerSEEK [30] similarly depend on limited genomic and proteomic panels, resulting in modest accuracy across a narrow range of cancer types. Recent integrative models (e.g., Bae et al. [31]) combine genomic and epigenomic signals using deep learning frameworks but require large reference cohorts and complex architectures and remain limited in cancer coverage. In contrast, our classifier integrates multidimensional, biologically informative cfDNA features derived from low-pass WGS, achieving competitive performance across 17 cancer types while maintaining cost-effectiveness and scalability. By incorporating microbial DNA, our model captures etiology-specific signals that may not be fully represented by host-derived features alone. Notably, it retains robust performance in clinically challenging low-TF samples, achieving top-1 and top-2 accuracies of 71% and 85%, respectively. This performance is in line with previously reported results in comparable low-signal cases; for instance, CUPid [13] correctly predicted 67.9% of samples with TF below 3%.

The predominantly high margin scores in single-tumor cohorts indicate confident top-1 predictions. In clinically challenging cases, such as low-TF and CUP cases, the top-2 prediction acts as a critical “diagnostic buffer”, ensuring the correct cancer origin remains within a narrowed differential diagnosis and reducing the risk of prematurely excluding potential sites. When margin scores are low or multiple predictions have similar probabilities, top-3 predictions may also be considered in conjunction with other clinical information to guide diagnostic decisions. Despite the inherent rarity and heterogeneity of CUP patients, our model correctly identified the primary site in 73–80% of cases, supporting a “liquid biopsy-first” workflow, where cfDNA-based TOO prediction can serve as an early triage tool alongside imaging, providing a high-confidence shortlist of potential origins. Clinicians can then prioritize specific IHC staining panels and targeted imaging (e.g., PET-CT or endoscopy), minimizing repeated, invasive biopsies and accelerating site-specific therapy.

Application of cfDNA-based TOO classification in patients with MPC is inherently more challenging due to the presence of mixed tumor-derived signals. Our exploratory analysis captured multiple tumor types among the top predictions, while reduced prediction confidence, reflected by wide margin score distributions, indicated comparable cfDNA contributions from multiple tumors or a dominant tumor. These patterns contrast with the clearer separation observed in single-tumor cohorts and underscore the limitations of single-label classifiers in multi-source biological contexts. Future approaches incorporating multi-label classification or cfDNA deconvolution may improve resolution in this setting.

Several limitations should be noted. First, performance was lower in certain cancer types, such as thyroid and prostate, likely due to limited representation in the training dataset. Second, subtype-level and metastatic-site-specific differences were not modeled because detailed annotations were unavailable during model development. Future studies with subtype-annotated cohorts could extend this approach to refine subtype-specific risk stratification and treatment guidance. Third, CUP and MPC cohorts were small and heterogeneous; therefore, these findings should be interpreted as exploratory and require validation in larger, more diverse cohorts. In particular, MPC cases may involve mixed cfDNA contributions from multiple tumor sources, highlighting the need for future models incorporating multi-label classification or cfDNA signal deconvolution.

In conclusion, this study demonstrates that integrated cfDNA profiling based on low-pass WGS can provide clinically informative TOO prediction across diverse cancer types. By combining complementary genomic, fragmentomic, methylation/repeat, and microbial signals, this approach offers a scalable non-invasive strategy to support diagnostic workup in settings where TOO assessment may inform clinical decision-making.

## Materials and methods

### Patient enrollment and plasma sample preparation

Blood samples were collected from multiple participating medical centers across China, including First Affiliated Hospital of Sun Yat-sen University, Zhongshan Hospital Fudan University, Xuzhou Medical University and its Affiliated Hospital, Sichuan Cancer Hospital & Institute, Jiangsu Cancer Hospital, Sun Yat-sen Memorial Hospital, Harbin Medical University Cancer Hospital, The First Affiliated Hospital of Guangzhou Medical University, Guangdong Provincial People’s Hospital, Nanjing Medical University, and Shandong Cancer Hospital and Institute. All samples were obtained from patients with histopathologically confirmed cancer prior to any therapeutic intervention and processed using a standardized protocol across all participating sites.

The study cohorts were constructed as follows. The training cohort was enrolled between February 1, 2022 and June 2, 2023 from the participating hospital network. The independent validation cohort was derived from the same network but collected during a temporally distinct and non-overlapping period from June 16, 2023 to August 31, 2024. No individual patient overlap existed between the two cohorts, and all samples were processed using identical pre-analytical and sequencing pipelines. Following initial quality control (QC), including exclusion of samples with insufficient cfDNA yield, degraded DNA integrity, or incomplete clinical annotation, 123 samples from the training cohort and 87 samples from the validation cohort were removed, leaving 1,814 samples in the training cohort and 1,221 samples in the validation cohort for analysis. Independent external cohorts for CUP and MPC were collected from dedicated clinical cases and were not part of the training or validation datasets. These cohorts were used exclusively for exploratory evaluation of model generalizability and were subjected to identical QC procedures as the primary cohorts.

For each participant, 10 mL of peripheral blood was collected in EDTA tubes and processed within 4 h. Plasma was extracted using a two-step centrifugation protocol: an initial centrifugation at 1,600 × g for 10 min at 4 °C, followed by centrifugation of the supernatant at 16,000 × g for 10 min at 4 °C. Plasma aliquots were stored at −80 °C and shipped on dry ice to the Central Laboratory of Nanjing Geneseeq Technology Inc. (Nanjing, China), a College of American Pathologists (CAP), the Clinical Laboratory Improvement Amendments (CLIA), and ISO 15189—certified laboratory. The study was approved by the Institutional Review Board (NSJB-MEC-2025–09), and written informed consent was obtained from all participants prior to sample collection.

### cfDNA extraction and whole-genome sequencing

cfDNA was extracted using the GENESEEQMag Cell-Free DNA Isolation Kit on an automated Hamilton Microlab STAR robotic liquid handler (Hamilton Company) to ensure consistency and minimize manual variability. Extracted cfDNA was quantified using the Qubit dsDNA HS Assay Kit (Thermo Fisher Scientific). For library preparation, 5–10 ng of cfDNA per sample was used for PCR-free library preparation with the KAPA Hyper Prep Kit (KAPA Biosystems) on the Biomek workstation (Beckman Coulter, Brea, CA). Low-pass whole-genome sequencing (WGS) was performed on the DNBSEQ-T7 platform (BGI, China) with 150 bp paired-end reads. To maintain analytical consistency, raw sequencing data were downsampled to a target depth of 5x. Samples with less than 3 × depth were excluded. Sequencing was performed across 17 batches for the training cohort and 11 for the validation cohort. Principal component analysis (PCA) confirmed no significant batch-driven variation (Fig. S7). Quality assurance included trimming low-quality reads with Trimmomatic [32], removing PCR duplicates using the Picard toolkit (http://broadinstitute.github.io/picard/), and aligning reads to the reference human genome GRCh37 with the Burrows-Wheeler Aligner [33]. Strict sample-level QC criteria were applied, including adapter dimer rate ≤ 0.005%, Q30 ≥ 95%, GC content 40–44%, clean base ratio ≥ 96%, mapping ratio ≥ 99%, insert size 150–200 bp, mean depth ≥ 5x, and duplicate rate ≤ 4%.

### Feature extraction

11 cfDNA profiling feature groups were extracted from low-pass WGS data to characterize cancer tissue-of-origin. Details on each feature extraction are described below:

#### Copy number variation (CNV)

CNV represents a type of genomic alteration characterized by gains or losses of DNA segments ≥ 1 kb. These CNV patterns reflect genomic instability and can highlight genomic regions frequently altered in different cancer types. In brief, the entire genome is divided into 1 Mb segments, which results in 2,475 bins. ichorCNA (version 0.3.2) [34] was used to profile copy number alterations. ichorCNA analyzes the observed read coverage data using a hidden Markov model, and the algorithm identifies regions with aberrant copy numbers. The log2 ratio (observed read count/expected read count) is calculated for each bin.

#### Repeat elements (RE)

Repeat DNA constitutes over 50% of the human genome [35]. Differences in RE representation can capture genome-wide structural patterns that vary across tissues and cancer types, providing informative signals for TOO prediction. A library of 24-bp short sequences (kmers), each uniquely occurring in one of the 1,266 repeat types across six families (LINE, SINE, LTR, RNA elements, satellites, and transposable elements). Additionally, kmers from 14 Human Satellite II and III subfamilies were included, bringing the total to 1,280 repeat types. For each sample, sequencing reads aligned with each kmer and its reverse complement were counted, and the kmer counts for each repeat type were aggregated. The aggregated counts were normalized to the number of reads that were aligned with mapping quality (MAPQ) to the reference human genome GRCh37.

#### Methylation-associated fragment ends (MFE)

Previous studies have shown that cfDNA fragments ending with N|CG motif are enriched at methylated CpG sites (N representing any nucleotide), whereas N|CCG-ending fragments are depleted due to competition with overlapping C|CG motifs [36]. These fragment end patterns reflect tumor-specific epigenetic modifications and chromatin organization. To capture these signals, we defined 12 genomic feature sets based on cancer-specific hypomethylated regions (hypo-DMRs) and repetitive elements. Ten of these feature sets were derived from MethMarkerDB (https://methmarkerdb.hzau.edu.cn/download/index), corresponding to the hypo-DMRs of 10 cancer types that overlap with targets of our study. The remaining two feature sets were defined based on repetitive DNA sequences, specifically Arthrobacter luteus (Alu) and Satellite regions, as these elements typically exhibit distinct hypomethylation signatures in malignancy and vary across cancer types [37, 38]. For each sample, the frequency of cfDNA fragment ends at N|CCG motif within these 12 regions was quantified as the ratio of fragments starting or ending at a specific N|CCG position to the total number of fragments overlapping a ± 50 bp window centered at that position.

#### Fragment size coverage (FSC)

Differences in cfDNA fragment lengths at a genome-wide scale reflect nucleosome positioning and chromatin accessibility, providing indirect information about the TOO. As previously described [39], the genome was partitioned into 5 Mb bins, resulting in a total of 541 intervals. For each interval, the count of short fragments (ranging from 100 to 150 bp) and long fragments (ranging from 151 to 220 bp) was determined. This yielded a total of 1082 counts across all bins. To ensure comparability and minimize bias due to varying fragment counts across intervals, the obtained counts were then scaled. The scaled count values were subsequently used as features for further analysis.

#### Fragment size distribution (FSD)

Fine-scale variations in cfDNA fragment sizes reflect nuclease cutting preferences and chromatin structure at higher resolution, which can vary across tissues and cancer types. Fragment coverage was measured in 5-bp intervals (100–104, 105–109, …, up to 215–219 bp) across 39 chromosome arms, as described previously [39, 40]. Counts were smoothed to reduce noise and then scaled to standardize values, generating features that capture detailed fragmentation patterns indicative of the cfDNA source.

#### Focal somatic copy number alterations (FocalCNV)

Focal CNVs highlight key genomic hotspots that are preferentially altered in specific tumor types, providing tissue- and cancer-type-specific signals useful for TOO inference. Somatic copy-number alterations (SCNAs) for 17 targeted cancer types were collected from TCGA datasets. A total of 652 distinct recurrent SCNAs regions, where copy-number alterations are most significant, were identified using GISTIC 2.0 [41], following the methodology described by Zack et al. [42]. Briefly, the SCNAs data were segmented to group continuous regions with similar copy-number alterations. These grouped regions, which show consistent alterations across a large number of tumor samples from different cancer types, were considered recurrent regions. The significance of these recurrent SCNAs regions was quantified using GISTIC 2.0, which identified regions with alterations that occur more frequently than expected by chance.

For the training cfDNA WGS data, preprocessing was performed as follows: BAM files were filtered to remove low-quality reads with mapping quality ≥ 15. GC correction was applied to adjust for GC content bias. Principal component analysis (PCA) was used for denoising the data. The baseline copy number distribution was defined using the average coverage depth of 1000 healthy individuals. The depth of the training cfDNA samples was then normalized relative to the baseline depth. The focal SCNA value for each region was computed using the log2 ratio of normalized depth between the sample and the baseline.

After preprocessing, differential testing was performed to identify regions with significant copy number alterations between the training data and the predefined 652 recurrent regions. The 18 most significant regions were selected as features for further analysis.

#### Nucleosome profiling (NP)

NP captures cfDNA fragmentation patterns that reflect nucleosome positioning and occupancy, providing indirect information on chromatin accessibility, transcriptional activity, and tissue-of-origin signals, all of which are often altered in cancer. Nucleosome occupancy around transcription factor (TF) binding sites was profiled using the Griffin framework. At open chromatin regions, nucleosomes are well-organized, allowing certain DNA segments to remain accessible to DNA-binding proteins. A loss of sequencing coverage indicates DNA protected by nucleosomes or other proteins, while peaks in coverage around these sites reflect degradation of unbound DNA. Briefly, a library of high mappability (≥ 0.95) binding sites for 854 TFs was compiled by integrating data from two sources. First, meta ChIP-seq data (version 19.10) were downloaded from the GTRD database (https://gtrd.biouml.org/downloads/19.10/chipseq/Homo sapiens_meta_clusters.interval.gz.) [43]. To ensure reliability, only TFs with known binding sites documented in the CIS-BP database (version 2.00, downloaded from http://cisbp.ccbr.utoronto.ca/bulk.php) were included [44]. The binding sites of each TF were filtered as follows: for TFs with more than 1000 sites, the top-1000 sites with the highest peak counts were selected; for those with fewer than 1000 sites, all sites were used. For each site in the library, sample sequencing read pairs with lengths between 100–200 bp from regions spanning −5000 bp and +5000 bp were extracted, after GC-content bias correction. The ±5000 bp window was split into 15 bp bins. Fragment midpoint counts were computed for each bin. Composite coverage profiles were then generated by averaging GC-corrected fragment midpoint coverages across all binding sites of each TF. From these profiles, three quantitative features were extracted: central coverage (±30 bp), mean coverage (±1000 bp), and nucleosome peak amplitude (calculated via Fast Fourier Transform analysis). Thus, a total of 2562 features were derived using the Griffin framework to assess chromatin accessibility and TF activity [45].

#### Orientation-aware cfDNA fragmentation (OCF)

Fragmentation patterns around open chromatin regions (OCRs) can capture orientation differences between upstream and downstream fragment ends. These orientation biases reflect nucleosome positioning and chromatin accessibility, providing indirect but highly specific information about the cell type and TOO [46]. The OCF score was calculated as described by Sun et al. [46]. To define the genomic coordinates for these features, we utilized publicly available pan-cancer ATAC-seq datasets from the NCI Genomic Data Commons (GDC; https://gdc.cancer.gov/about-data/publications/ATACseq-AWG). From the 23 primary cancer types reported in the GDC publication, we selected 13 cancer-specific OCR sets that overlapped with our study targets. Additionally, one T-cell-specific OCR set was included to account for immune-derived cfDNA contributions, resulting in a total of 14 OCF feature variables.

For each OCR, a 120 bp window was defined, centered on the nucleosome-depleted region with 60 bp flanking on each side. To compute OCF scores, cfDNA fragment end positions were extracted from each sample’s paired-end WGS data. Fragment ends were aggregated into two categories relative to the OCR center: upstream ends (from −60 bp to the center) and downstream ends (from the center to +60 bp). The OCF score was then calculated by measuring the divergence between upstream and downstream end signals in 20 bp windows around the positions of highest read density relative to the center as follows:

$$\mathrm{O}\mathrm{C}\mathrm{F} = {\sum}_{-60-10}^{-60+10}(D-U)+ {\sum}_{60-10}^{60+10}(U-D)$$

#### SBS-based mutational signatures (SBS)

SBS mutational signatures capture DNA damage and repair processes characteristic of different cancer types, providing insights into tumor-specific genomic processes and TOO. Raw sequencing data in FASTQ format were first trimmed using Trimmomatic [32] and aligned with the reference human genome GRCh37 using BWA mem (version 0.7.15). Duplicate reads were removed, and the aligned reads were sorted and indexed into BAM files by SAMtools (version 1.9) [47]. The average sequencing depth was calculated from the resulting BAM files. Low-quality reads were excluded from analysis based on the following criteria: unpaired reads, base quality score < 30, mapping quality score < 40, alternative alignments, or fragment length > 300 bp. Genomic regions with repetitive or low-complexity sequences were masked prior to variant calling. To identify single base substitutions (SBSs), paired-end reads were scanned for single mismatches at the central position of a 3-nucleotide window, ensuring that the substitution was concordant between R1 and R2 reads. Insertions and deletions were excluded. To focus on somatic mutations, SBSs were further filtered against known germline variants in the gnomAD (version 2.0) and an internal panel of normals generated from healthy individuals. Each validated SBS was then classified into one of 96 different nucleotide contexts based on the 6 possible substitutions (C > A, C > G, C > T, T > A, T > C, and T > G) with their two flanking bases on each side. Counts were normalized by mean sequencing depth to adjust for coverage differences. The 96-bin mutational context matrix was fitted to COSMIC SBS signatures (version 3.3) using the MutationalPatterns package (version 3.16) to get the final contributions of each SBS signature to the mutational profile of the sample [48].

#### Microsatellite instability (MSI)

MSI reflects defects in DNA mismatch repair, which is prevalent in cancers such as colorectal, endometrial, and gastric tumors. MSI was assessed using MSIsensor-pro [49], a robust computational tool for MSI detection that does not require matched normal samples. For each sample, sequencing data were analyzed against a predefined list of stable microsatellite sites. At each microsatellite site, MSIsensor-pro models the distribution of observed repeat length by a multinomial distribution. Sites are classified as unstable if their observed allelic distribution significantly deviates from the expected distribution derived from stable samples. For each sample, the MSI score was calculated as the proportion of unstable sites, defined as the number of unstable sites divided by the total number of evaluated microsatellite sites.

#### Microbial DNA sequences

The presence of microbial DNA can reflect viral or bacterial infections that are intrinsically linked to oncogenesis and may influence multiple cancer hallmarks [50, 51]. To capture these signals, raw sequencing reads in FASTQ format were first aligned to the reference human genome GRCh37 to remove host-derived sequences. Reads that failed to align were subsequently mapped to a microbial reference database using Kraken for taxonomic classification [52]. The abundance of six target microbial species was subsequently quantified with Bracken [53], including *Hepatitis B virus* (HBV)*, Human gammaherpesvirus 4* (EBV)*, Human gammaherpesvirus 8* (HHV-8)*, Helicobacter pylori, Alphapapillomavirus 9* (primarily α9 and α7, encompassing HPV-16 and HPV-18, respectively), and *Mycobacterium tuberculosis*.

### Machine learning and model development

A summary of input variables categorized by feature type is provided in Table S3. The final tissue-of-origin classifier was developed using all features after normalization and the H2O AutoML framework on the training cohort, with five-fold cross-validation to optimize performance. A total of 100 base models were generated across five algorithm families, including Deep Learning (*n* = 34), Distributed Random Forest (DRF, *n* = 2), Gradient Boosting Machine (GBM, *n* = 27), Generalized Linear Model (GLM, *n* = 1), and XGBoost (*n* = 36), as specified by the AutoML parameter max_models = 100. Each base model performs multi-class classification, outputting a probability for each cancer type per sample, with the sum of probabilities equal to one. Deep learning models implement a feedforward neural network, with hyperparameters provided in Supplementary Material 3.

The predictions from these base models were then used to train meta-learners, automatically generating 15 stacked ensemble models. The ensembles included two types: BestOfFamily, which combines the best-performing base model from each algorithm family, and AllModels, which combines all base models regardless of algorithm type. In total, 115 models (100 base models +15 ensemble models) were evaluated on the training set based on top-1 accuracy, and the model with the highest training accuracy was selected as the final classifier (Fig. S8; Table S4; Supplementary Material 4). For prediction, the cancer type with the highest probability is reported as the top-1 prediction, while the second-highest probability is reported as the top-2 prediction. The top-2 prediction provides additional plausible candidate tissue-of-origin predictions, which can be informative for clinically challenging samples.

### Statistical analysis

Statistical analyses were performed using R (v4.5.1) and Python (v3.11). Categorical variables were compared using the chi-square test or Fisher’s exact test, as appropriate, and continuous variables were compared using the Wilcoxon rank-sum test. Model performance was evaluated using multiple metrics, including accuracy, sensitivity (recall), precision, area under the receiver operating characteristic curve (AUROC), and F1-score. For multi-class classification, both macro-averaged and weighted-averaged metrics were reported. Macro-averaged metrics were calculated by computing each metric independently for each class and then taking the unweighted mean, thereby treating all classes equally. In contrast, weighted-average metrics were computed by weighting each class-specific metric by its sample proportion, reflecting overall performance in the presence of class imbalance. Top-1 accuracy was defined as the proportion of samples for which the highest predicted probability corresponded to the true cancer type, while top-2 accuracy was defined as the proportion of samples for which the true cancer type was among the two highest predicted probabilities. All P-values were two-sided, and *P* < 0.05 was considered statistically significant.

## Supplementary Information

Supplementary Material 1.

Supplementary Material 2.

Supplementary Material 3.

Supplementary Material 4.

## Data Availability

The code and datasets utilized and/or analyzed in the current study are available from the corresponding author upon reasonable request.
