# Supplementary material for: Integrative cfDNA profiling from low-pass whole-genome sequencing enables tissue-of-origin prediction in cancer
Source: Mol Biomed. 2026 Aug 5;7:125. doi: 10.1186/s43556-026-00497-2 (PMC13437874; doi:10.1186/s43556-026-00497-2)
Supplement: Supplementary file 1 [file 43556_2026_497_MOESM1_ESM.docx]

**Supplementary Material 1**

**Manuscript title: Integrative cfDNA Profiling from Low-Pass Whole-Genome Sequencing Enables Tissue-of-Origin Prediction in Cancer**

Yunjian Zhang^1,†^, Liang Liu^2,†^, Hua Bao^3,†^, Ke Xu^3^, Hao Zhang^4,5^, Song Wang^3^, Shuang Chang^3^, Dongqin Zhu^3^, Zongyao Huang^6^, Zheng Wang^7^, Liu Yang^8^, Bingzhong Zhang^9^, Ji Tao^10^, Wenhua Liang^11^, Jierong Chen^12^, Shanshan Yang^3^, Xue Wu^3^, Yang Shao^3,13,^*, Wenquan Wang^2,^*, Dongyuan Zhu^14,^*

^1^Department of Breast Surgery, First Affiliated Hospital of Sun Yat-sen University, Guangzhou, China

^2^Department of Pancreatic Surgery, Zhongshan Hospital, Fudan University, Shanghai, China

^3^Geneseeq Research Institute, Nanjing Geneseeq Technology Inc., Nanjing, China

^4^Thoracic Surgery Laboratory, Xuzhou Medical University, Xuzhou, China

^5^Department of Thoracic Surgery, Affiliated Hospital of Xuzhou Medical University, Xuzhou, China

^6^Department of Pathology, Sichuan Clinical Research Center for Cancer, Sichuan Cancer Hospital & Institute, Sichuan Cancer Center, University of Electronic Science and Technology of China, Chengdu, China

^7^Department of Liver Surgery and Transplantation, Liver Cancer Institute, Zhongshan Hospital, Fudan University, Shanghai, China

^8^Colorectal Center, Jiangsu Cancer Hospital, Nanjing, China

^9^Department of Gynecologic Oncology, Sun Yat-sen Memorial Hospital, Sun Yat-sen University, Guangzhou, China

^10^Department of Gastrointestinal Medical Oncology, Harbin Medical University Cancer Hospital, Harbin, China

^11^Department of Thoracic Surgery and Oncology, The First Affiliated Hospital of Guangzhou Medical University, Guangzhou, China

^12^Department of Clinical Laboratory, Guangdong Provincial People’s Hospital, Guangzhou, China

^13^School of Public Health, Nanjing Medical University, Nanjing, China

^14^Department of Rare Tumors, Shandong Cancer Hospital and Institute, Shandong First Medical University and Shandong Academy of Medical Sciences, Jinan, China

**^†^These authors contributed equally to this paper**

***Co-corresponding authors**

**Dongyuan Zhu** ([zhudy@sd-cancer.com](mailto:zhudy@sd-cancer.com)); **Wenquan Wang** ([wang.wenquan@zs-hospital.sh.cn](mailto:wang.wenquan@zs-hospital.sh.cn)); **Yang Shao** ([yang.shao@geneseeq.com](mailto:yang.shao@geneseeq.com))

## **Supplementary Tables**

| **Table S1. Model performance for top-1 predictions across 17 individual cancer types in the training cohort** | | | | | | | | | | |
| --- | --- | --- | --- | --- | --- | --- | --- | --- | --- | --- |
| **Cancer type** | **Total** | **TP** | **FP** | **FN** | **Accuracy** | **Precision** | **Sensitivity** | **F1** | **AUROC** | **AUROC_95 CI** |
| Breast | 125 | 88 | 15 | 37 | 0.704 | 0.854 | 0.704 | 0.772 | 0.976 | 0.968-0.985 |
| Cervical | 97 | 76 | 20 | 21 | 0.784 | 0.792 | 0.784 | 0.788 | 0.990 | 0.985-0.995 |
| Colorectal | 137 | 120 | 19 | 17 | 0.876 | 0.863 | 0.876 | 0.870 | 0.992 | 0.987-0.996 |
| Esophagus | 76 | 58 | 25 | 18 | 0.763 | 0.699 | 0.763 | 0.730 | 0.970 | 0.950-0.990 |
| Gastro | 155 | 132 | 24 | 23 | 0.852 | 0.846 | 0.852 | 0.849 | 0.980 | 0.968-0.992 |
| Head_Neck | 101 | 89 | 6 | 12 | 0.881 | 0.937 | 0.881 | 0.908 | 0.987 | 0.975-0.998 |
| Kidney | 76 | 60 | 28 | 16 | 0.789 | 0.682 | 0.789 | 0.732 | 0.971 | 0.953-0.989 |
| Liver_Biliary | 194 | 163 | 31 | 31 | 0.840 | 0.840 | 0.840 | 0.840 | 0.976 | 0.963-0.989 |
| Lung | 160 | 114 | 36 | 46 | 0.713 | 0.760 | 0.713 | 0.735 | 0.931 | 0.909-0.954 |
| Melanoma | 50 | 37 | 8 | 13 | 0.740 | 0.822 | 0.740 | 0.779 | 0.942 | 0.895-0.989 |
| Ovary | 127 | 107 | 14 | 20 | 0.843 | 0.884 | 0.843 | 0.863 | 0.990 | 0.984-0.997 |
| Pancreas | 107 | 91 | 22 | 16 | 0.850 | 0.805 | 0.850 | 0.827 | 0.968 | 0.943-0.994 |
| Prostate | 111 | 68 | 24 | 43 | 0.613 | 0.739 | 0.613 | 0.670 | 0.966 | 0.951-0.981 |
| Sarcoma | 86 | 65 | 28 | 21 | 0.756 | 0.699 | 0.756 | 0.726 | 0.978 | 0.966-0.991 |
| Thyroid | 59 | 31 | 17 | 28 | 0.525 | 0.646 | 0.525 | 0.579 | 0.940 | 0.909-0.970 |
| Urothelium | 80 | 67 | 33 | 13 | 0.838 | 0.670 | 0.838 | 0.744 | 0.980 | 0.963-0.996 |
| Uterus | 73 | 53 | 45 | 20 | 0.726 | 0.541 | 0.726 | 0.620 | 0.978 | 0.970-0.986 |
| Abbreviations: TP, true positive; FP, false positive; FN, false negative; AUROC, area under the receiver operating characteristic curve; CI, confidence interval | | | | | | | | | | |

| **Table S2. Model performance for top-1 predictions across 17 individual cancer types in the validation cohort** | | | | | | | | | | |
| --- | --- | --- | --- | --- | --- | --- | --- | --- | --- | --- |
| **Cancer type** | **Total** | **TP** | **FP** | **FN** | **Accuracy** | **Precision** | **Sensitivity** | **F1** | **AUROC** | **AUROC_95 CI** |
| Breast | 80 | 58 | 10 | 22 | 0.725 | 0.853 | 0.725 | 0.784 | 0.971 | 0.951-0.990 |
| Cervical | 63 | 52 | 18 | 11 | 0.825 | 0.743 | 0.825 | 0.782 | 0.991 | 0.985-0.996 |
| Colorectal | 94 | 81 | 15 | 13 | 0.862 | 0.844 | 0.862 | 0.853 | 0.982 | 0.967-0.996 |
| Esophagus | 52 | 39 | 12 | 13 | 0.750 | 0.765 | 0.750 | 0.757 | 0.969 | 0.935-1 |
| Gastro | 105 | 89 | 12 | 16 | 0.848 | 0.881 | 0.848 | 0.864 | 0.973 | 0.955-0.992 |
| Head_Neck | 71 | 64 | 9 | 7 | 0.901 | 0.877 | 0.901 | 0.889 | 0.989 | 0.970-1 |
| Kidney | 56 | 45 | 11 | 11 | 0.804 | 0.804 | 0.804 | 0.804 | 0.968 | 0.936-1 |
| Liver_Biliary | 131 | 112 | 26 | 19 | 0.855 | 0.812 | 0.855 | 0.833 | 0.968 | 0.950-0.987 |
| Lung | 108 | 82 | 26 | 26 | 0.759 | 0.759 | 0.759 | 0.759 | 0.969 | 0.954-0.984 |
| Melanoma | 34 | 24 | 5 | 10 | 0.706 | 0.828 | 0.706 | 0.762 | 0.959 | 0.919-0.999 |
| Ovary | 82 | 72 | 9 | 10 | 0.878 | 0.889 | 0.878 | 0.883 | 0.987 | 0.975-1 |
| Pancreas | 72 | 58 | 10 | 14 | 0.806 | 0.853 | 0.806 | 0.829 | 0.980 | 0.965-0.995 |
| Prostate | 74 | 55 | 18 | 19 | 0.743 | 0.753 | 0.743 | 0.748 | 0.978 | 0.967-0.990 |
| Sarcoma | 57 | 43 | 12 | 14 | 0.754 | 0.782 | 0.754 | 0.768 | 0.984 | 0.973-0.994 |
| Thyroid | 38 | 21 | 11 | 17 | 0.553 | 0.656 | 0.553 | 0.600 | 0.931 | 0.886-0.977 |
| Urothelium | 54 | 44 | 17 | 10 | 0.815 | 0.721 | 0.815 | 0.765 | 0.954 | 0.916-0.992 |
| Uterus | 50 | 36 | 25 | 14 | 0.720 | 0.590 | 0.720 | 0.649 | 0.984 | 0.977-0.991 |
| Abbreviations: TP, true positive; FP, false positive; FN, false negative; AUROC, area under the receiver operating characteristic curve; CI, confidence interval | | | | | | | | | | |

| **Table S3. Summary of input variables categorized by feature type for the final model** | |
| --- | --- |
| **Feature Name** | **Count** |
| Copy number variation (CNV) | 2475 |
| Repeat elements (RE) | 1280 |
| Methylation-associated fragment ends (MFE) | 12 |
| Fragment size coverage (FSC) | 1082 |
| Fragment size distribution (FSD) | 936 |
| Focal somatic copy number alterations (FocalCNV) | 18 |
| Nucleosome profiling (NP) | 2562 |
| Orientation-aware cfDNA fragmentation (OCF) | 14 |
| SBS-based mutational signatures (SBS) | 175 |
| Microsatellite instability (MSI) | 1 |
| Microbial DNA sequences | 6 |

| **Table S4. Model specifications for the selected stacked ensemble model** | |
| --- | --- |
| **Parameter** | **Value** |
| model_id | StackedEnsemble_AllModels_5_AutoML_1_20250603_183743 |
| metalearner_algorithm | xgboost |
| metalearner_nfolds | 5 |
| metalearner_fold_assignment | NA |
| fold_assignment | NA |
| keep_levelone_frame | TRUE |
| blending_frame | NA |
| training_frame | NA |
| validation_frame | NA |
| seed | 9.1857E+18 |
| score_training_samples | 10000 |
| score_validation_samples | NA |
| n_base_models | 100 |
| Note: NA (not applicable) indicates parameters or configurations that were not explicitly set or utilized in this specific model architecture. | |

## **Supplementary Figures**

### **Figure S1. Supervised dimensionality reduction of integrated cfDNA features reveals cancer type-specific separation patterns**

**(a, b)** Linear discriminant analysis (LDA) was applied to visualize class-specific patterns across cancer types in the training (a) and validation (b) cohorts using all 11 cfDNA features. Each point represents one sample and is colored by cancer type.

### **Figure S2. Exploratory evaluation of individual and integrated cfDNA feature performance for TOO identification**

Performance comparison of ten individual cfDNA feature categories (excluding MSI) and the integrated multi-feature model (“All features”) in the training cohort (**a**) and independent validation cohort (**b**).

### **Figure S3. Confusion matrices for the PCS predictor in the training cohort stratified by tumor fraction**

Confusion matrices illustrating multi-class classification performance across 17 cancer types in the training cohort, stratified by tumor fraction and prediction rank. (**a**) High tumor fraction samples, top-1 prediction; (**b**) High tumor fraction samples, top-2 prediction; (**c**) Low tumor fraction samples, top-1 prediction; (**d**) Low tumor fraction samples, top-2 prediction. Rows represent predicted classes and columns represent actual classes. Cell values indicate the number of samples, with color intensity reflecting the proportion of predictions. Diagonal values correspond to correctly classified samples. Per-class sensitivity is indicated on the top horizontal axis, and per-class precision is indicated on the right vertical axis.

### **Figure S4. Confusion matrices for the PCS predictor in the validation cohort stratified by tumor fraction**

Confusion matrices illustrating multi-class classification performance across 17 cancer types in the validation cohort, stratified by tumor fraction and prediction rank. (**a**) High tumor fraction samples, top-1 prediction; (**b**) High tumor fraction samples, top-2 prediction; (**c**) Low tumor fraction samples, top-1 prediction; (**d**) Low tumor fraction samples, top-2 prediction. Rows represent predicted classes and columns represent actual classes. Cell values indicate the number of samples, with color intensity reflecting the proportion of predictions. Diagonal values correspond to correctly classified samples. Per-class sensitivity is indicated on the top horizontal axis, and per-class precision is indicated on the right vertical axis.

### **Figure S5. Model performance across clinical stages and tumor fraction levels in the validation cohort**

Bar plots illustrate the performance of the PCS predictor across clinical stages, stratified by tumor fraction (TF) levels. Multiple evaluation metrics are shown: the top row presents macro-averaged accuracy, AUROC, F1-score, precision, and sensitivity, while the bottom row shows the corresponding weighted-averaged metrics.

### **Figure S6. Distribution of cancer-associated microbial reads and MSI scores across cancer types**

(**a**) Box plots showing normalized read counts for representative microbial species across 17 cancer types. (**b**) Distribution of microsatellite instability (MSI) scores across all cancer types.

### **Figure S7. Assessment of potential batch effects in cfDNA sequencing data**

Principal component analysis (PCA) was performed on sequencing data from the training cohort (**a**) and the validation cohort (**b**) to evaluate potential batch effects. Each point represents a sample, colored by sequencing batch.

### **Figure S8. Performance evaluation of machine learning algorithms for model selection**

Box plot showing the distribution of performance metrics across 115 trained models, grouped by algorithm type: Distributed Random Forest (DRF), Generalized Linear Model (GLM), Deep Learning, Gradient Boosting Machine (GBM), XGBoost, and Stacked Ensemble. Each dot represents an individual model. The final model was selected based on the highest training accuracy among all candidates to ensure optimal classification performance.
